# Design and Performance Evaluation of a Low-Cost Autonomous Sensor Interface for a Smart IoT-Based Irrigation Monitoring and Control System

**DOI:** 10.3390/s19173643

**Published:** 2019-08-21

**Authors:** Sani Abba, Jonah Wadumi Namkusong, Jeong-A Lee, Maria Liz Crespo

**Affiliations:** 1 Department of Mathematical Sciences (Computer Science), Faculty of Science, Yelwa Campus, Abubakar Tafawa Balewa University (Federal University of Technology), Dass Road, P.M.B. 0248, Bauchi, Nigeria; 2 Computer Systems Laboratory, Department of Computer Engineering, Chosun University, Dongku SeoSukDong 375, Gwangju 501-759, Korea; 3 Multi-disciplinary Laboratory (MLab), Abdussalam International Centre for Theoretical Physics (ICTP), Via Beirut 31, 34014 Trieste, Italy

**Keywords:** autonomous sensor interface, internet of things (IoT), soil moisture, smart irrigation system, monitoring and control, fabricated prototype, low-cost, Arduino integrated design environment, soil moisture sensor

## Abstract

Irrigation systems are becoming increasingly important, owing to the increase in human population, global warming, and food demand. This study aims to design a low-cost autonomous sensor interface to automate the monitoring and control of irrigation systems in remote locations, and to optimize water use for irrigation farming. An internet of things-based irrigation monitoring and control system, employing sensors and actuators, is designed to facilitate the autonomous supply of adequate water from a reservoir to domestic crops in a smart irrigation systems. System development lifecycle and waterfall model design methodologies have been employed in the development paradigm. The Proteus 8.5 design suite, Arduino integrated design environment, and embedded C programming language are commonly used to develop and implement a real working prototype. A pumping mechanism has been used to supply the water required by the soil. The prototype provides power supply, sensing, monitoring and control, and internet connectivity capabilities. Experimental and simulation results demonstrate the flexibility and practical applicability of the proposed system, and are of paramount importance, not only to farmers, but also for the expansion of economic activity. Furthermore, this system reduces the high level of supervision required to supply irrigation water, enabling remote monitoring and control.

## 1. Introduction and Related Works

### 1.1. Introduction

Today, farmers usually work on large portions of land that are partitioned to grow different types of crops. During the dry season, they practice irrigation farming. There is a shortage of water for irrigation, and it is not possible for a person to monitor the amount of water content in the soil, in order to keep the root of the plant moist, or to detect it in real time. This study aims to address the water shortage problem, that is often faced by farmers using irrigation systems, by providing an autonomous sensor interface for the remote monitoring and control of the supply of water to the soil, thereby removing much of the effort required by farmers.

Recently, the use of intelligent sensory techniques has gained a significant amount of attention by farmers who practice irrigation agriculture. These techniques have been applied in agriculture to plan numerous activities and tasks appropriately, by utilizing limited resources with less human intervention. Aeroponics is a modern agricultural method that is commonly practiced around the world. In this system, plants are cultivated under complete control conditions in a growth chamber, by way of a light misting of a nutrient solution, replacing the soil medium. These nutrient mists are ejected through atomization nozzles on a periodic basis. During the plant cultivation period, many parameters are optimized, including temperature, humidity, light intensity, water nutrient solution level, pH and EC levels, CO_2_ concentration, atomization time, and atomization interval time, in order to enhance plant growth [1].

With the present technological advancements, there is a greater demand for specialized information regarding agricultural practices. This is because much of agricultural studies are based on exploratory investigations, on a local scale, using ground data by employing a single remote sensor. In addition, the methods employed mostly depend on local knowledge of management practices, environment, and biological materials. These limitations call for a new direction toward smart irrigating [2].

A scheme for the optimal watering of agricultural crops, using a wireless sensor network, has been previously presented by the authors [3]. In that study, the main goal was to design and develop a control system using a sensor node in the crop field, with data management and application control, through a smart phone and web application interface. The proposed scheme provides three components: (1) The hardware control box, (2) web-based application, and (3) mobile application. In addition, the system allows either, automatic, or manual control, by the user. The results showed the usefulness of this system in agriculture, by reducing cost and increasing agricultural productivity.

The use of internet of things (IoT) technology for agricultural practices has been demonstrated in monitoring citrus soil moisture and nutrients, the integration of fertilization and an irrigation decision support system. Significant achievements have been made. These include, a single-point, multi-layer citrus soil temperature and humidity detection, wireless sensor node and citrus precision fertilization, and the construction of an irrigation management decision support system. This approach can help farmers improve the use of fertilizers in the irrigation system. In addition, it increases the precision of citrus production, and reduces labor costs and pollution, caused by the application of chemical fertilizers to the soil [4].

In a smart irrigation system, the design and fabrication of a self-powered and autonomous fringing-field capacitive sensor, used to measure soil water content, is possible. This kind of sensor is manufactured using a conventional printed circuit board (PCB) and incorporates a porous ceramic material. The design allows the energy-harvesting module to operate, based on the condition that the micro-sprinkler spinner irrigates the soil, and the supercapacitor is fully charged to 5 V in approximately three hours during the first irrigation. Subsequently, with the supercapacitor fully charged, the system can supply power from the supercapacitor for approximately 23 d, without any energy being harvested [5].

Agricultural practices have been the most important means of living over the course of human evolution. Hence, humans depend on a wide range of agricultural products in nearly every facet of life. The process involved in the artificial application of water to farmlands, in assisting the growth of plants, is referred to as irrigation agriculture. This process has become a great advantage in complementing agricultural activities, especially in the dry season. Irrigation plays a vital role in advancing the agricultural, educational, and economic growth of the nation as a whole. The traditional method of farming is undertaken manually, by engaging individual farmers in almost all stages of crop development and monitoring and control of the processes involved at all stages of irrigation farming. From time-to-time, these processes are replaced with semi-automated and automated processes, to provide assistance to farmers and improve agricultural productivity. The four traditional irrigation practices are ditch irrigation, drip irrigation, terraced irrigation, and the use of a sprinkler system. Owing to the increased demand for agricultural raw materials for industrial use and human consumption, irrigation tends to attract a significant amount of attention, particularly in the developed and underdeveloped world. In the traditional system, the farmers apply water to plants mechanically at different stages of plant development, to assist in the growth and development lifecycle. However, an inadequate supply of water to plants affects plant growth, and the scarcity of water has become a global issue, particularly in the irrigation system, which is specifically caused by global warming. Various human activities, including the burning of fuel, industrial activities, and deforestation have been the major causes of the depletion of the ozone layer and inadequate rainfall, particularly affecting plant growth and development. The traditional approach to irrigation, involves the use of watering cans and water channels, which must be manually monitored and controlled. This causes a significant amount of water to be wasted and misused. Therefore, an autonomous sensor interface, for a smart IoT-based irrigation system, must have components that both monitor and control the water levels available to plants, without failure or the need for human intervention [6].

Agricultural practices use 85% of the available freshwater resources worldwide. The high demand and consumption of water, owing to rapid human population growth and agricultural practices are the dominant factors of water consumption. Accordingly, urgent attention is required to create techniques and strategies, based on science and technology, in order to maintain and sustain the use of water for agricultural development. The idea of an autonomous sensor interface for an IoT-based irrigation monitoring and control system results from plant watering management requirements. Hence, modern Internet technology is essential for resolving the challenges of the limited supply of water to farms, and providing better lasting solutions for irrigation management and control. A wireless sensor network (WSN) can be used in conjunction with IoT for a smart irrigation system. Therefore, the use of WSN can provide the means of communication, computation, and sensing information from near and remote places. The combination of IoT and sensor network technology facilitates the proper use and management of water resources for a smart irrigation system. This will help boost smart agricultural practices and produce higher crop yields annually [7,8].

The salient contributions of this article are as follows:

1. A low-cost autonomous sensor interface is proposed to determine whether watering is required for plants, based on the information obtained from the monitoring and control of the soil water content.
2. A monitoring and control system (implemented prototype) that supplies the amounts of water, required by plants to avoid drying, is designed.
3. The system should stop the water supply when the required amount has been supplied to plants.
4. A convenient sensor-based interface architecture and web portal are provided so that the user can monitor the system remotely.

This study is of paramount importance, not only to farmers that practice irrigation farming, but also to the expansion of economic growth in general. This method encourages irrigation farming by reducing the high levels of supervision required by farms to ensure the supply of water to farms, by using wireless sensor network and IoT technology. The rest of the article is organized as follows: Section 2 discusses the system design methodology; Section 3 discusses the system implementation; Section 4 presents the experimental results and discussion; and Section 5 provides the concluding remarks and describes future work.

### 1.2. Related Works

Many studies have contributed toward a smart irrigation system. The high demand for food requires rapid improvement in food production technology. Several researchers have made significant efforts to find a solution for irrigation farming. However, these efforts have yet to provide an efficient solution to the problems of the present irrigation system.

The review paper by Imran et al. [1] contributes significant knowledge regarding early fault detection and diagnosis in Aeroponics, using wireless sensor networks. The Aeroponics technique allows the farmer to monitor several parameters, without using laboratory instruments, and the farmer has the ability to control the entire system remotely. In addition, this technique provides valuable information, essential for plant researchers and a greater understanding of how the key parameters of Aeroponics relate to plant growth in the system.

The review paper by Agnés et al. [2] provides an extensive study of remote sensing for crop mapping practices. The authors emphasize that there is a need to have specialized information regarding agricultural practices. The study is categorized into three groups of practices: Crop succession, cropping pattern, and cropping techniques. The authors observed that the majority of studies were based on exploratory investigations, tested on a local scale, using ground data by employing a single remote sensor. In addition, the methods employed mostly depend on local knowledge, based on management practices, environment, and biological materials. These limitations can be used to identify future research directions, which include the use of land stratification, multi-sensor data combination, and expert knowledge-driven methods. The authors concluded by proposing that new spatial technologies, particularly the sentinel constellation, are anticipated to advance the monitoring of cropping practices in the context of food security, and to provide the best administration of agro-environmental issues. An interesting system was presented for the optimal watering of agricultural crops, which employ the use of a wireless sensor network [3]. The proposed system provides the capabilities of manual and automatic control of crops by the application user.

ZigBee technology, artificial intelligence, and decision support technology have also been employed [4] to monitor the moisture content of citrus soil. This study provided insight into the application of IoT for agricultural practices in the monitoring of citrus soil moisture and nutrients, and the integration of fertilization, as well as an irrigation decision support system. Remarkable advancements were made, including single-point multi-layer citrus soil temperature and humidity detection, wireless sensor nodes and citrus precision fertilization, and an irrigation management decision support system. The results of their study indicated that the system can help a farmer improve the use of fertilizers in an irrigation system. In addition, the system increases the precision of citrus production, and reduces the labor cost and pollution caused by the application of chemical fertilizers to the soil.

The design and fabrication of a self-powered and autonomous fringing field capacitive sensor, to measure soil water content, was presented [5]. The sensor used was manufactured using a conventional PCB and in which a porous ceramic material was incorporated. To obtain the sensor reading, the authors used a circuit containing a 10-kHz triangle wave generator, AC amplifier, precision rectifier, and micro-controller. This approach developed a complete irrigation control system that integrates the sensor, an energy-harvesting module, composed of a micro-generator installed on top of a micro-sprinkler spinner, and a DC/DC converter circuit that charges a 1-F supercapacitor. The design allows the energy-harvesting module to operate on the condition that the micro-sprinkler spinner irrigates the soil, and the supercapacitor is fully charged to 5 V, in approximately 3 h, during the first irrigation. Subsequently, with the super-capacitor fully charged, the system was capable of supplying power from the super-capacitor for approximately 23 d, without any energy being harvested.

The Yogesh et al. study [6] demonstrates that the watering system controller sends transitory information. It presents the output from the sensor and presents the possibility of being programmed to enhance system performance. The moisture sensor connects to devices via a remote network and transmits the sensed information, within a time interval, synchronized to the Internet, to provide guidelines on how the system is to be operated. The soil moisture sensor is controlled by the watering system if the solenoid valve is in an open state. These two components are the major building blocks of irrigation system control. Therefore, the systematic control of the irrigation field should be adopted. For instance, the moisture sensor should operate in a controlled range, e.g., when the moisture value is lower or higher than the set threshold value, the solenoid valve controls the operation by opening, or closing the valve, respectively.

The Arduino board was utilized in [7,8], which consists of an ATmega 328 microcontroller. The microcontroller is programmed in such a way as to sense the moisture level of the plants. The design was undertaken to supply the required amount of water to the plants. The system is utilized for plant maintenance and monitoring of the entire farm land. Thus, the plants can be watered twice daily, in the morning and the evening. Hence, the use of a microcontroller plays a vital role in the watering and monitoring of the plants on a daily basis.

Marie et al. [9] reported that the sensed data, regarding the quantity of water content level in the soil, that was obtained from a moisture sensor, can significantly improve the agricultural yield. This would motivate researchers to perform extensive studies in various fields of interest that are related to the irrigation systems. A significant advancement in measuring the development of plant growth is the minimization of the costs of irrigation management, particularly for a mechanized watering system. This approach would significantly reduce water wastage and lessen the amount of human power required for the continuous monitoring of the source of water supply in an irrigation system. The use of a feedback-based system would efficiently help track and manage resources, compared with open-loop systems, that are at the expense of higher complexities. Thus, soil moisture is difficult to correctly measure, and the target moisture content levels are difficult to sustain.

The continuous increase in the demand for agricultural products and raw materials has necessitated a rapid improvement in the development of food technology [10]. Therefore, only agricultural practices can provide the solution to this problem. The use of an automated irrigation system has triggered the continuous demand for agricultural farm products. The available water resources are scarce, and the inadequacy of land and water has resulted in a decrease in the volume of water worldwide. Farmers normally employ an irrigation system during the dry season. In irrigation agriculture, two important aspects must be considered: Information on the fertility of the soil, and; the content of soil moisture. Famers have employed different methods and techniques in an irrigation system to reduce the dependence on rainfall as a major source of water supply to the soil. The most commonly used technique is the use of electrical energy, combined with an on-and-off scheduling control system to power the irrigation system. Other available techniques operate based on atmospheric climate conditions. The use of smart microcontrollers, used to sense the climatic conditions and determine the supply of water in real-time, is an important technique for a smart irrigation system. This approach employs the following: Internet-based monitoring using servers; general packet radio service modem; global system for mobile communication; and wireless monitoring using Bluetooth and WSNs.

Many farmers require financial aid to innovate their agricultural practices. Numerous master frameworks have been provided to assist farmers boost agricultural productivity. Nevertheless, these primary frameworks depend on a base-learning approach. Therefore, a master system framework, that integrates IoT technology, is highly recommended, as it will exploit the continuously generated real-time data [11].

Gabriel et al. [12] emphasized that the majority of farmers cannot afford to use sophisticated technologies for agricultural practices. It is now of paramount importance that more effective, cheaper solutions need to be implemented. The study proposed building virtual organizations of agents, that can communicate between each other, while monitoring crops in a farmland. Simple and low-cost sensor architecture helps farmers monitor and optimize the growth of their crops, by restructuring the amount of resources the crops require at each stage of their development. The approach employs the use of Platform for automatic coNstruction of orGanizations of intElligent agents (PANGEA) architecture to overcome the limitations of hardware and processing capabilities. The designed system is capable of collecting heterogeneous information from its environment using sensors for temperature, solar radiation, humidity, pH, moisture, and wind. The main finding of their approach is that the solution can merge heterogeneous data from sensors and produce a response adapted to each situation. The authors presented a case study to validate the proposed system. However, its drawback is that it is not economically feasible to use a TV screen to monitor the condition of crops in this context. Further, the monitored data are presented in real time, which may affect the monitoring of the crop moisture level, thereby resulting in poor crop yield.

The design of SmartFarmNet, which is an IoT-based platform that provides the services of collection of environmental, soil, fertilization, and irrigation data, was presented in [13]. The system autonomously correlates such data, and filters out invalid data from the viewpoint of evaluating crop performance, providing forecasting information, and formulating recommendations for a specific farmland. The proposed SmartFarmNet possesses the capability of integrating virtually any IoT device, including commercially available sensors, cameras, and weather stations, and has the ability to store the captured data in the cloud for performance analysis and recommendations. In conclusion, SmartFarmNet was presented as the first and, presently, the largest system in the world to offer crop performance analysis and recommendations.

Chandan and Pramitee [14] presents a smart irrigation system. The system is cost effective and affordable by middle-class farmers. The 21st century is characterized by automation technology that plays an important role in human life. This technology allows devices to be controlled automatically to provide comfort, reduce energy consumption, increase efficiency and save time. The machine automation and control used by the modern industries are not cost effective and cannot be used by the local farmers. In contrast, their work presents a low-cost irrigation system that can be afforded by Indian farmers. The paper’s main objective is to automatically control the water motor that selects the direction of water flow in a pipe with the aid of soil moisture sensor. The information regarding the operation of the motor, and the direction of the water flow in the farmland, is sent by SMS to the users’ mobile phones.

Karthikeswari and Mithraderi [15] designed a low-cost automated irrigation system using wireless sensor network and GPRS module. The work was aimed at reducing the manual monitoring of the farmland and using GPRS technology to provide information to users. The system can determine whether free electricity supply is being used or electric motors are used for pumping water to the farmland. This will prevent misuse of the electricity supply to pumping mechanisms. And hence, reduces water wastage.

A review of irrigation system and economical and generic automatic irrigation system, using GSM-Bluetooth for irrigation remote monitoring system was presented by Purnima and Reddy [16]. The proposed system possesses the features of low cost, low power consumption for remote monitoring and control of sensors, using SMS and GSM module. The proposed system notifies the users about any abnormal circumstance that may arise. These include lower moisture content, temperature rise, CO_2_ concentration etc. The information regarding these parameters is sent to the Famer’s mobile phone via GSM or Bluetooth modules for appropriate action.

Archana and Priya [17] presents an automatic plant watering system. The research work focuses on the water scarceness and the need for a smart and efficient way of irrigation. The authors implemented a humidity sensor to detect the soil humidity in the agricultural farmland. The system supplies the adequate water requirement to the irrigated farmland. The authors employed the use of microcontroller to supply water to the irrigation field. Sensors are buried in the soil to sense the water content of the soil. The sensors are activated when there is a presence, or no presence, of water in the field. Once the sensor identifies dry soil, it activates the microcontroller and pump water to the field.

Ferrarezi et al. [18] developed automated irrigation controllers that employ the use of capacitance sensors and data loggers to supply water to plants on demand. The limitation of the proposed approach was the cost implications, due to data loggers and software programs, to build and control the system controllers. However, the use of low-cost open-source microcontrollers provides a better way to build a sensor-based irrigation controller for irrigation agriculture and domestic use. The authors built an automated irrigation system employing microcontroller, capacitance moisture sensors, and solenoid valves. The proposed system efficiently monitored and controlled the volumetric water content thresholds of (0.2, 0.3, 0.4, and 0.5 m^3^·m^−3^) with Panama red hibiscus (Hibiscus acetosella) in a peat substrate. The system operates both on regular 24 V alternating current solenoid valves and latching 6 V to 18 V direct current solenoid valves. The technology employed has an average cost. The microcontroller used and accessories cost $107, four capacitance soil moisture sensors cost $440, and four solenoid valves cost $120. The total cost an estimated value of $667 US Dollars. However, our proposed approach is more cost effective, and is only valued at $79 USD. Other approaches are the automatic irrigation based on monitoring plant transpiration, hyperspectral remote sensing for detecting soil salinization using ProSpecTIR-VS aerial imagery and sensor simulation, and high temperature AlGaN/GaN membrane-based pressure sensors are provided by the respective authors in [19,20,21].

This study attempts to address the limitations of the above-mentioned approaches by their respective authors. Accordingly, this work could identify the following limitations, which we intend to rectify: (1) the use of sprinklers as a means of water supply to the soil; (2) the use of a semi-automated system, which does not monitor or control the soil moisture content, and the lack of descriptive analysis to assist the farmer in tracking the moisture content level of the soil on the Internet; and (3) a low-cost and flexible design architecture for real-time monitoring and control of soil moisture content.

## 2. Autonomous Sensor Interface System Architecture and Design

### 2.1. Autonomous System

An autonomous system is defined as a system that detects its operating environment and senses the operating parameters, changes its operating behavior in that environment, and adapts to the changes and events occurring in that environment. Autonomic systems provide the capabilities to solve system complexities by using technology to manage and control dynamical systems. These types of systems operate with independent and pre-defined conditions, protocols, and policies, without human intervention. Autonomous systems can configure, optimize, manage, and control their actions according to predetermined conditions, rules, and learning the environments in a given period of time. The origin of the word autonomic comes from the field of human biology. For example, the human body consists of an autonomic nervous system that monitors and controls the human heartbeat, check blood sugar levels, and regulates and maintains body temperature to normal without human intervention. An autonomous sensor interface can be employed in smart irrigation agriculture to solve the problem of water shortage to the plants. Autonomous systems provide the capabilities of self-managing its operation by using appropriate actions in response to situational changes that arise in a dynamic environment. The main objective of an autonomous system is to monitor and control the loop that generates and collects detailed information from the surroundings and supply the appropriate action. As outlined in [22,23], an autonomous system has four characteristics as follows:

1. Self-Configuration: This is the ability of the autonomous system to adapt dynamically to environmental and situational changes, based on pre-defined protocols, and principles that govern the operating environment. Self-configuration provides continuous signal strength in sensing environmental parameters and enhances sensor-node productivity. This will provide scalability and flexibility in the sensor network interface.
2. Self-Healing: This feature provides the means of autonomous discovery and fault detection. It provides the ability to discover, diagnose, and respond to unpredicted situational changes that may occur in a dynamic environment. Self-healing detects sensor component failure and initiates a repair technique without affecting system workability. The system repairing mechanisms makes it resilient against errors and faults.
3. Self-Optimization: This feature provides autonomous monitoring and control to facilitate the fair and optimal use of limited resources. Self-optimizing sensor components possess capabilities of controlling themselves to attain the stated goals and objectives in a given environment. The optimization techniques are the reallocation and rescheduling of resources caused by arising changes in the dynamic environment, to enhance the overall sensor network utilization, real-time transmission, and retransmission of sensor data.
4. Self-Protection: This feature provides the capabilities of anticipating, identifying, and providing mechanisms for protection against threats and various kinds of attacks in autonomous systems. This feature enables the autonomous system to detect malicious and suspicious behavior within the system and take reactive measures to counterattack them and make the system less vulnerable to threats. Furthermore, this feature provides security measures and policies to protect components and the entire system against all internal or external threats and vulnerability attacks.

In summary, these four characteristics of autonomous systems are employed in the design and implementation of an autonomous sensor interface for IoT-based irrigation monitoring and control systems for agricultural use. The system can configure, heal, optimize, and protect its operation, and enhance the supply of water to the plant to increase agricultural production.

### 2.2. Autonomous Sensor Interface System Architecture

**Figure 1:**
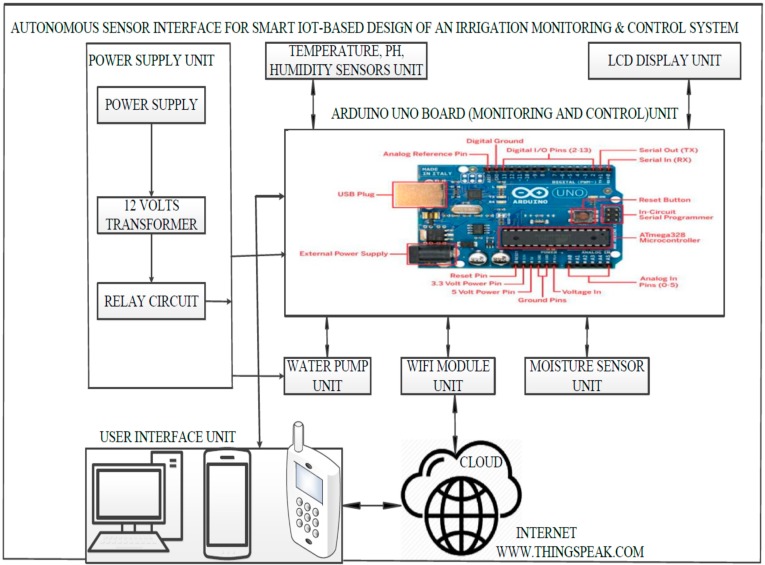
Low-cost sensor interface for smart IoT-based irrigation monitoring and control system architecture.

The low-cost autonomous sensor interface for the design of smart irrigation system architecture consists of the input and output processes, as illustrated in Figure 1. The entire system operation is controlled by the Arduino Uno board, which is programmed on the behavior of other system components [24]. The system consists of the following eight units: Power supply; water pump; WiFi module; soil moisture sensor; Arduino board; temperature, pH, and humidity sensors; user interface; and a 16 × 2 liquid crystal display (LCD) unit. The combination of these components makes the integration of the system easier to debug and provides optimal performance. The sensor values are uploaded on the Internet at 5-min intervals. The power supply unit provides a continuous supply of electricity to the smart irrigation system; it utilizes a 12 V transformer and a relay circuit. The system uses a generic AC 220 V to 12 V DC step down power supply module for Arduino generic PCs to power the board and the water Pump. A 12 V relay circuit is used to regulate and control the water pump. The board can be supplied with power, either from the DC power jack (7–12 V), the USB connector (5 V), or the VIN pin of the board (7–12 V). The power output is supplied to the Arduino board and the water pumping unit. The water pumping unit provides a mechanism by which adequate water in due proportion is supplied to the plants. This mechanism is programmed, controlled, and monitored by the Arduino Uno board. The Arduino board unit is the main monitoring and control unit. The Arduino Uno is a microcontroller board based on the ATmega328. It has 14 digital input/output pins, 6 analog inputs, a 16-MHz ceramic resonator, and a universal serial board. This unit is programmed using embedded C language to provide the necessary control signals to manage the entire irrigation system. The moisture sensor unit is responsible for measuring the soil moisture content of the water level of the soil, using the sensor uses capacitance to measure the water content of the soil. This is essential in smart agricultural practices, as it helps farmers monitor and control the smart irrigation system. In practice, this sensor, which is flexible and configurable, is inserted into the irrigation farmland i.e., soil to be examined and the volumetric water content of the soil is reported in percentage. The user interface unit provides a convenient interface to access and programme the sensors. The system users have the ability to interact with the system, using desktop, laptop, and smartphone devices. The LCD unit is a medium through which the microcontroller communicates with the user. The LCD unit is used to display sensed data readings from the sensors. The WiFi module unit is a self-contained system-on-chip (SoC) device with an integrated transmission control protocol/internet protocol (TCP/IP) stack, that provides access to a WiFi network. It has the capability of hosting an application or offloading all WiFi networking functions from another application processor. The module is a wireless local area network (WLAN) module with devices, based on IEEE standards. Furthermore, WiFi-compatible devices can connect to the Internet via a WLAN and wireless access point [25,26].

### 2.3. Real-Time System Monitoring and Control

**Figure 2:**
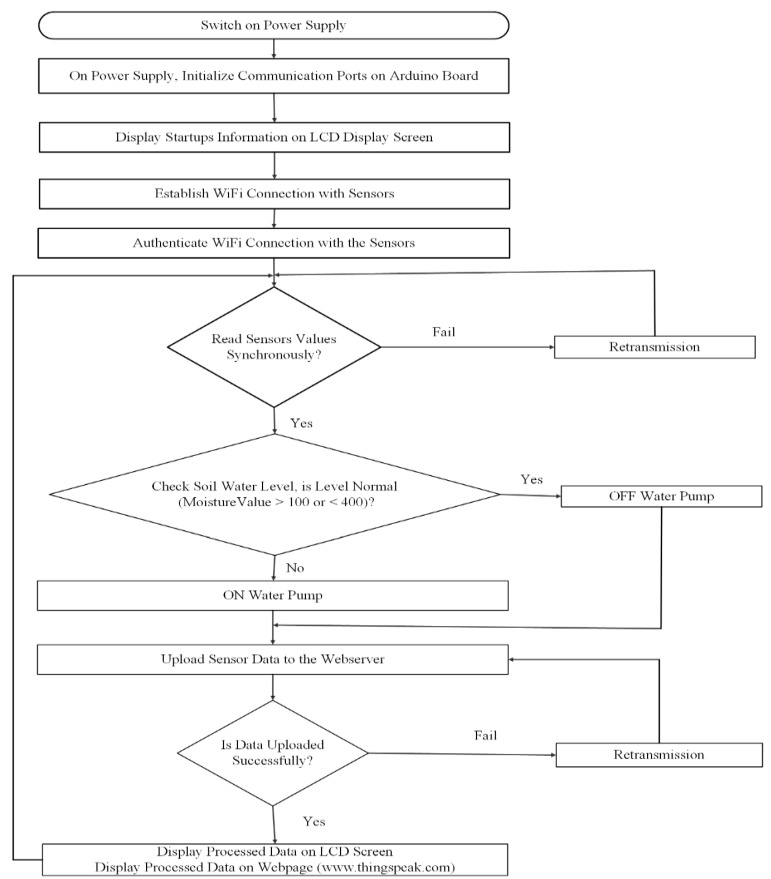
Real-time system monitoring and control.

Figure 2 illustrates the real-time device monitoring and control system. The connected sensor components and ports are initialized to display the start-up information on the 16 × 2 LCD screen. The microcontroller subsequently attempts to establish connection with the different components of the system. As soon as the connection is established, the WiFi module and moisture sensors will authenticate the established connection and start reading the moisture content level of the soil. The readings are displayed on the LCD screen through the user interface window of the Arduino serial monitor. In situation whereby the sensor readings are not obtained, i.e., there is no established connection, a retransmission is possible. The real-time water level (moisture sensor) module is accessed through the Internet at the www.thingspeak.com interface to obtain the monitoring values from the sensors. The date and time when the data was sensed are recorded. The two sensors autonomously obtain the measurement of the indicated parameters, such as temperature, humidity, and moisture level. The recorded measurements are saved in the Internet server at thingspeak.com, along with the date and timestamp. After the recorded data are received, a descriptive chart is plotted to indicate the moisture level of the soil. The system is programmed to switch the pump ON and OFF, based on the soil moisture content, using the embedded C programming language, as illustrated in Figure 2.

### 2.4. System Modeling and Simulation

**Figure 3:**
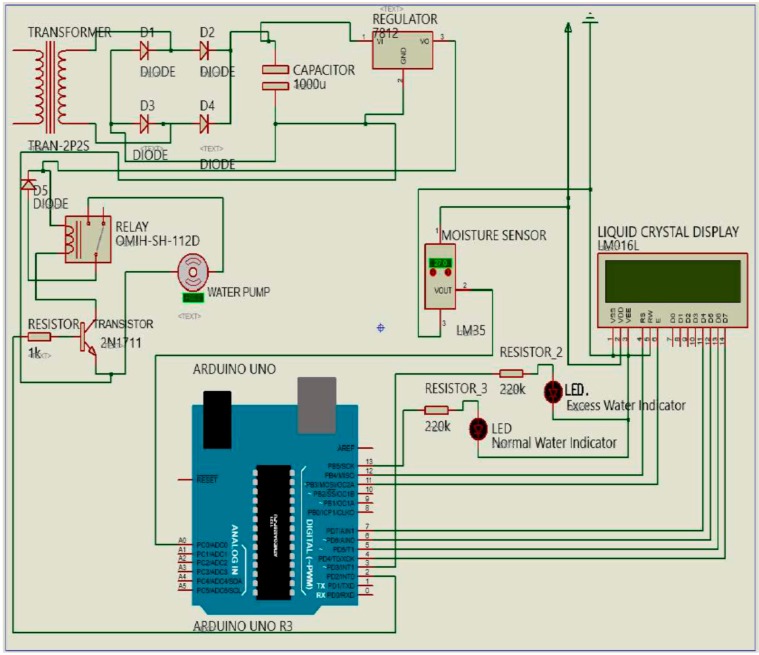
Schematic diagram of a smart irrigation modeling and simulation system.

The low-cost sensor interface, for the smart irrigation system, is simulated using the Proteus 8.5 design suite, Arduino Uno integrated development environment (IDE), and embedded C programming language. Proteus 8.5 Professional is an efficient tool and a high-performance design environment for simulating technical computing [25]. It integrates computation and the virtual representation of the systematic operation of the system, displaying how the constructed system components will work. Arduino, as the development board, provides an IDE, by which source codes can be written, verified, and uploaded to the microcontroller. Arduino is an open-source electronics platform that provides hardware/software co-design tools for designing embedded applications [24]. The embedded C programming language is suitable for the design of embedded systems and sensor components. The codes, written in embedded C language, allow the circuit or devices to achieve their optimal performance. In addition, it has a failure rate of less than one percent when developed to interact with hardware components. The Proteus 8.5 is a professional PCB design tool that can be integrated with a shape-base auto router; it provides features, such as fully featured schematic capture, highly configurable design rules, and interactive SPICE circuit simulation. Proteus design combines the ISIS schematic capture and ARES PCB layout programs to provide a powerful, integrated, and easy-to-use tool suite for educational and professional PCB design. Figure 3 illustrates the basic schematic components of the autonomous sensor interface for the smart IoT-based irrigation system. The moisture sensor, 16 × 2 LCD, transformer, light-emitting diode (LED), diode, Arduino Uno (microcontroller), water pump, power regulator, relay circuit, and many other devices constitute a complete irrigation monitoring and control system. Another approach is the use of a field-programmable gate array and system-on-chip for the monitoring and control system [26,27,28].

### 2.5. The Moisture Sensor Calibration

Herein, we present the sensor calibration technique, used in this study. A two-point calibration method was employed [29,30,31] to calibrate the moisture sensor input. The Arduino board is configured to takes sensor readings simultaneously for 5 s during the system startup. The system then monitors the highest and lowest values of the sensor readings. These sensor readings, taken during the first five seconds, are termed the minimum and maximum of the anticipated values for the readings taken during the system loop. A two-point calibration technique is applied to the raw moisture sensor outputs. This approach is very important to re-scaling the output and has the potential to correct both slope and offset errors. Pseudocode 1 shows the procedure used to calibrate the moisture sensor, mentioned in the experimental section. The following configuration needs to be set before the Arduino startup process. The initial values for the minimum and maximum of the moisture sensor are set to *int MoistureMin =* 400*,* and *int MoistureMax =* 100, respectively.

The configuration above looks surprising. However, it calibrates the output of the sensor. First, the MoistureMin is set high (400) and monitored for any reading below that, saving it as the new minimum. Similarly, the MoistureMax is set low (100) and monitored for any reading higher, as the new maximum. In doing so, any further moisture readings can be mapped to the range between the MoistureMin and MoistureMax values. Finally, the moisture sensor can be calibrated using the map function, as illustrated in Algorithm 1.


**Algorithm 1: Soil moisture sensor calibration process.**
1: // Algorithm to Calibrate the moisture sensor during the first five seconds. 2: // Set the initial values for the minimum and maximum values of the moisture sensor. 3: ***int***
*MoistureMin* ← 400; // The minimum moisture sensor value. 4: ***int***
*MoistureMax* ← 100; // The maximum moisture sensor value. 5: ***while*** (millisecond () < 5000) 6:   { 7:   *MoistureValue* ← ***analogRead*** (MoisturePin) 8:   // Record the maximum sensor value 9:   ***if*** (*MoistureValue* > *MoistureMax*) 10:      { 11:        *MoistureMax* ← *MoistureValue* 12:      } 13:   // Record the minimum sensor value 14:    ***if*** (*MoistureValue* < *MoistureMin*) 15:    { 16:      *MoistureMin* ← *MoistureValue* 17:     } 18:   } 19: *MoistureValue* ← ***map*** (*MoistureValue*, *MoistureMin*, *MoistureMax*, 100, 400);

### 2.6. The Soil Moisture Sensor

There are two classes of sensors that measure the soil moisture content. The first class is known as, the soil moisture sensors, and this class of sensors measure the volumetric water content in soil. The second class is referred to as soil-water potential sensors. This class of sensor measures another property of moisture in soils called the water potential, including tensiometers and gypsum blocks. Meanwhile, the direct gravimetric measurement of free-soil moisture requires removing, drying, and weighing of a soil sample. While the soil moisture sensors measure the volumetric water content indirectly, by employing any other property of the soil, such as dielectric constant, resistance, capacitance, or interaction with neutrons as a substitute for the moisture content [32].

The research by Jim Bilskie [33] is based on the direct gravimetric and volumetric measurements of soil water status content and potential. As mentioned by the author, the state of water in soil is defined in relation to the amount of water and energy associated with the forces, which hold the water in the soil. The amount of water, that is described by the water content and the energy state of the water, is the water potential.

The soil-water content can be expressed as direct or indirect on a gravimetric or volumetric basis. The direct gravimetric water content ($\Phi_{g}$) is the mass of water per mass of dry soil. It is measured by weighing a soil sample (*Mass_Wet*), drying the sample to remove the water, then weighing the dried soil (*Mass_Dry*). This is given in Equation (1):

(1) $$\Phi_{g}\;=\;\frac{Mass\_Water}{Mass\_Soil}\;=\;\frac{Mass\_Wet\;-\;Mass\_Dry\;}{Mass\_Dry}$$

The direct volumetric water content $\Phi_{v}$ is the volume of liquid water per volume of soil. Volume is the ratio of mass to density (ρ), this is given in Equation (2).

(2) Φv= Volume_WaterVolume_Soil = Mass_Waterρ_WaterMass_Soilρ_Soil = Φg × ρ_Soilρ_Water

Another parameter for the direct volumetric water content is the soil bulk density (ρ_*Bulk*), which is used for soil density (ρ_*Soil*) parameter, and is defined as the ratio of soil dry mass to sample volume. Herein, the density of water is close to 1 and most situations is ignored. Furthermore, another important property of the soil is the soil porosity (*ε*), this property has a direct relationship with the soil bulk density, as provided in Equation (3). Where the parameter ρ_Solid is the density of the soil fraction and is approximated and given as 2.6 g·mc^−3^:

(3) $$\varepsilon \;=\;1\;-\;\frac{\rho \_Bulk}{\rho \_Solid}$$

The soil moisture sensor module, used in this study, is based on the LM393 comparator. Farmers need to measure the soil moisture regularly to prevent plants from drying. Hence, measuring the soil moisture is very important in agriculture to help farmers remotely manage and control the irrigation system. This type of sensor indirectly measures the volumetric water content of the soil. To measure the soil moisture content, the moisture sensor uses the capacitance to measure the water content of soil. The operating principle, used by the soil moisture sensor, uses the capacitance to measure dielectric permittivity of the surrounding environment. In soil, dielectric permittivity is a function of the water content. The soil moisture sensor generates a voltage proportional to the dielectric permittivity, and consequently the water content of the soil. The moisture sensor averages the water content over the whole length of the sensor. In the sensor, there exists a 2 cm zone of influence, with respect to the flat surface of the sensor. However, there is little or no sensitivity at the extreme edges of the flat surface. The soil moisture sensor is practically applied to quantify the loss of moisture for a dedicated period of time, due to evaporation and plant uptake. It evaluates the optimum soil moisture content for various species of plants, and monitors soil moisture content to control irrigation in agricultural farmland. The sensor can be inserted into the soil to be tested, and the volumetric water content of the soil is reported as a percentage. In doing so, the farmer can precisely monitor and control the amount of water to be supplied, with the aid of an easy-to-use mechanism, using a water pump and a microcontroller unit. The conductivity of the soil may be severely altered by the application of fertilizers, and this would result in false soil moisture readings when using a conductive probe.

**Figure 4:**
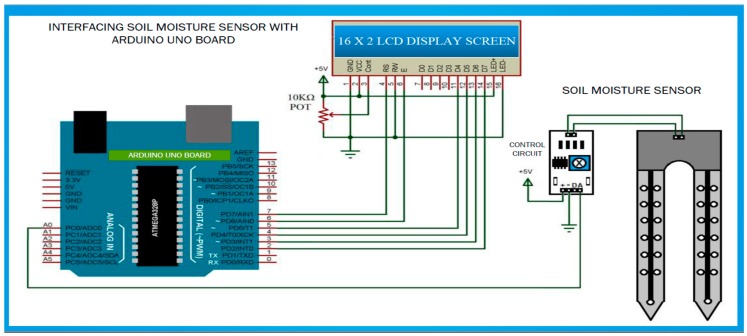
Low-cost sensor interface for smart irrigation modeling and simulation system.

**Figure 5:**
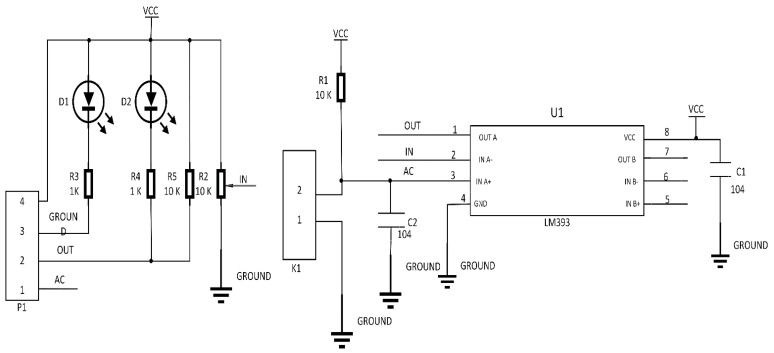
Circuit diagram of the moisture soil sensor operating principle.

The soil moisture sensor comprises two parts: The main sensor and the control board unit. The main sensor part consists of a number of conductive probes, that can be used to measure the volumetric content of the water in the soil. The control unit is designed, based on the LM392 IC, as a voltage comparator. Other components included are connectors, LEDs, resistors, and capacitors. Furthermore, a potentiometer is provided to adjust the sensitivity of the control unit module, as illustrated in Figure 4. The ground moisture sensor operates based on the principle of voltage comparison, as exemplified in Figure 5. Herein, one input of the comparator is connected to a 10-kΩ potentiometer, and the other input is tied to a voltage divider network, designed with a 10-kΩ resistor and a sensor probe for insertion into the ground. The probe conductivity varies, depending on the water content. In a situation by which the water content of the soil is less, the probe conductivity would be less; therefore, the input to the comparator would be HIGH. This implies that the output of the comparator is HIGH; based on this, the LED would be in the OFF state. Furthermore, in a situation in which there is adequate water content in the soil, the probe conductivity increases, resulting in a comparator output of LOW. Thus, the LED would start to blink. An essential feature of this sensor module is that it provides an analog output that can be sent to the microcontroller. The analog signal can be transmitted to the analog input pin of the Arduino board. The volumetric water content of the soil can be measured and presented as a percentage [34,35].

## 3. System Implementation

**Figure 6:**
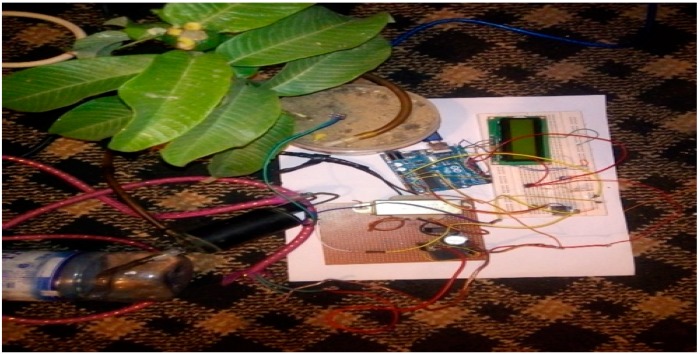
Low-cost sensor interface for Internet of Things (IoT)-based irrigation system component setup.

**Figure 7:**
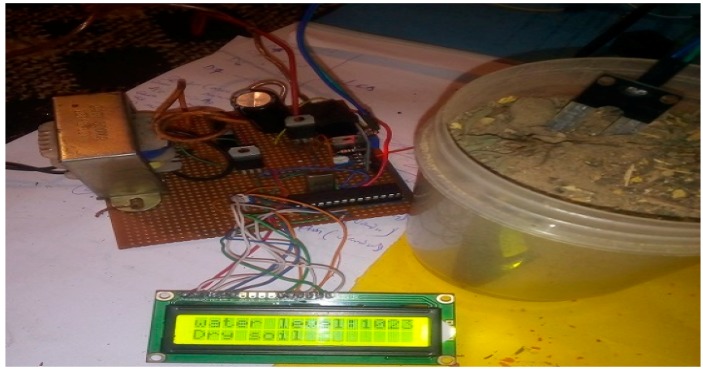
Low-cost sensor interface for IoT-based irrigation system sensor probe inserted in completely dry soil.

**Figure 8:**
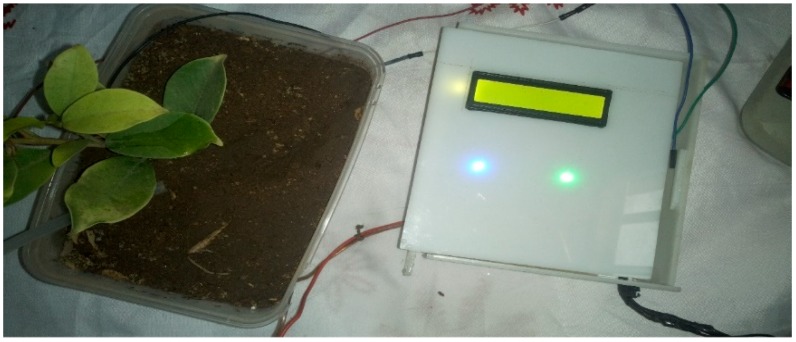
Prototype testing in less moist soil (Moisture level 25%).

**Figure 9:**
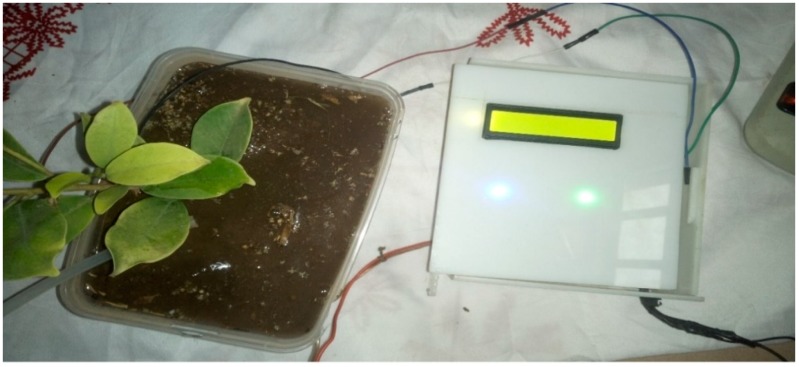
Prototype testing in highly moist soil (Moisture level 410%).

**Figure 10:**
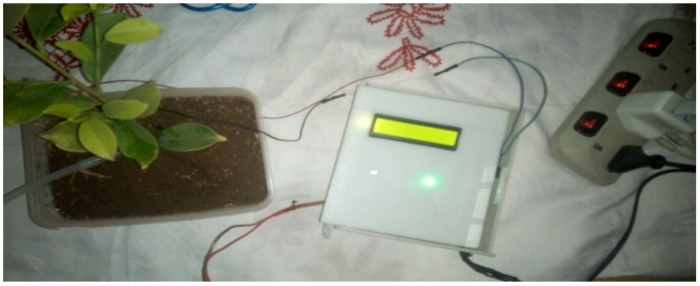
Prototype testing in less dry soil (Moisture level 17%).

**Figure 11:**
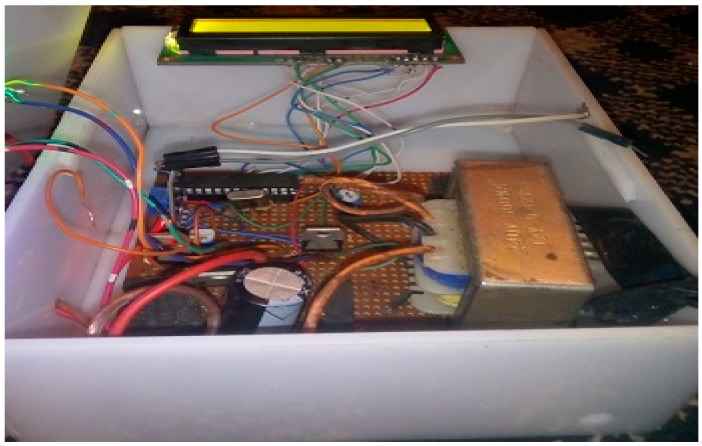
The implemented prototype view part 1.

**Figure 12:**
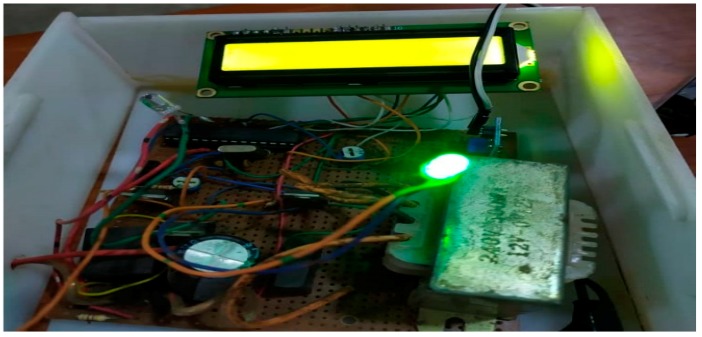
The implemented prototype view part 2.

The implementation of the low-cost autonomous sensor interface, for a smart IoT-based irrigation monitoring and control system, establishes that the new system is operational and satisfies the operational requirement of the user for measurement and evaluation. The important parameters to be measured, by the smart system, have been defined and are well-established. The moisture sensors are buried in the ground at the required depth of 2 cm. Once the soil reaches the desired moisture level, the sensors send a signal to the microcontroller to turn off the relay circuits, which control the water pump. The implementation can be tested in the designated location. These tests include, arranging the constructed system prototype at a strategic location in farmland, such that the environmental parameters would be monitored by the sensors. The processed sensor data are transmitted to the web server through the website, www.thingspeak.com [27], where users can view the monitoring and control data. Figure 6, Figure 7, Figure 8, Figure 9 and Figure 10 illustrate the experimental setup of the autonomous sensor interface for the IoT-based irrigation system. The components are interconnected via a smart interface, the water pump is connected to the plants to supply water when the moisture level of the soil is below normal, and the immersed moisture sensor, buried in the soil, monitors the moisture level and communicates the information to the microcontroller. Thereafter, the monitored and control information is transmitted to the LCD screen, and uploaded to the web server, through the WiFi module. Figure 11 and Figure 12 demonstrate the implemented prototype.

## 4. Experimental Results and Discussion

The experimental results are obtained from the implemented prototype. We conducted several experiments under different simulated scenarios to demonstrate the workability of the proposed system. The results are the outputs obtained from the calibrated sensor readings, transmitted and stored on the website, www.thingspeak.com. The prototype was operated and tested to evaluate device workability in real situations. The date and timestamp of the data of each sensor are displayed on the website for user information.

### 4.1. Sensor Data Logging

**Figure 13:**
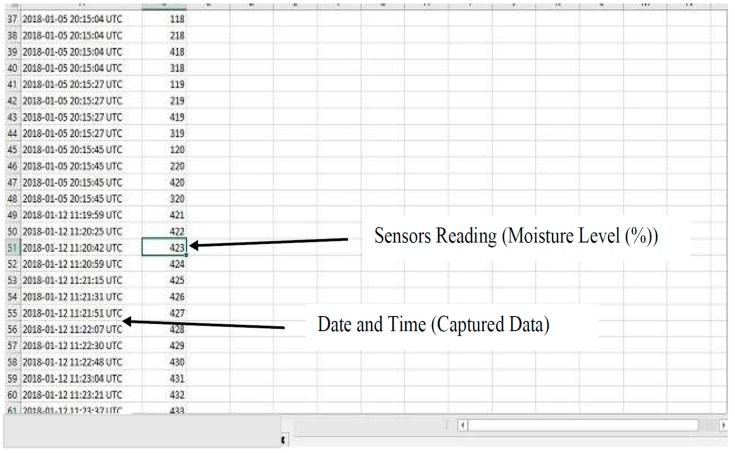
Sensor data logging.

The calibrated data, captured by the sensors, are transmitted to the web server [27]. The moisture sensor sends data, through the WiFi module to www.thingspeak.com, is accessible via a phone or personal computer. Here, the logged data of the moisture content values, with the appending date and time, are shown in the serial terminal, as illustrated in Figure 13. The numerical data can also be downloaded and saved in comma-separated values (CSV) or the excel file format. It is important to obtain the moisture content values, in order to demonstrate the efficiency and data integrity of the developed system prototype. This represents significant advances in irrigation system technology, particularly in the present age of serious advancements in sensor technology and creativity.

**Figure 14:**
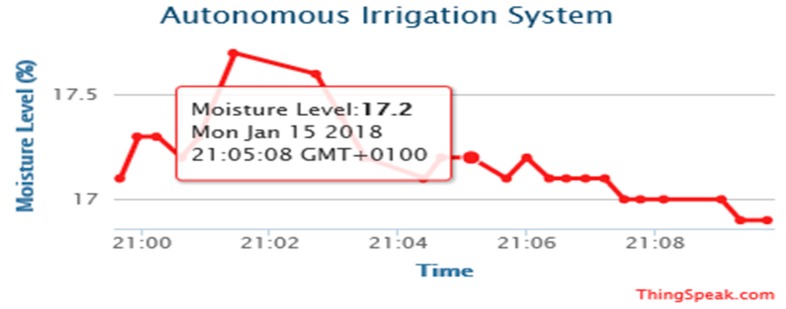
Moisture levels against time obtained from http://www.thingspeak.com (API key =9VHO8NR618JJT9CN).

**Figure 15:**
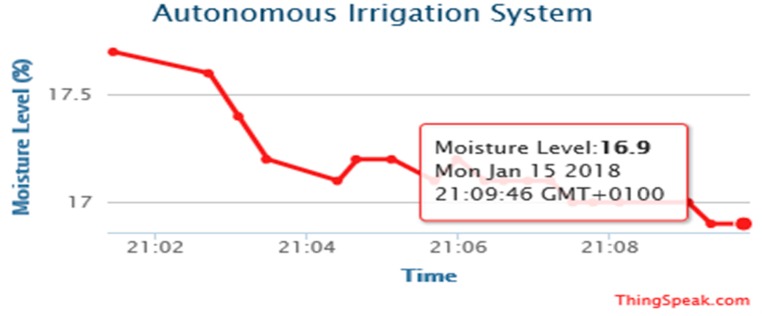
Moisture levels against time obtained from http://www.thingspeak.com (API key = FE4H530DNA2NJQXB).

**Figure 16:**
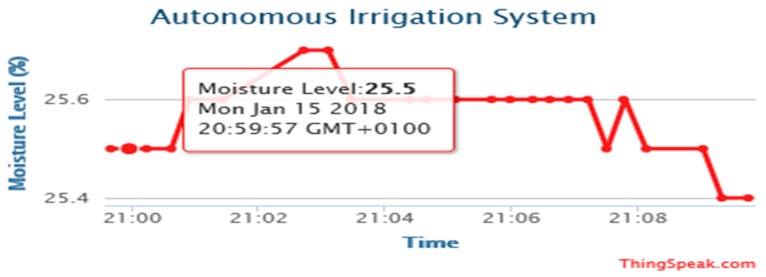
Moisture levels against time obtained from http://www.thingspeak.com (API key = FE4H530DNA2NJQXB).

**Figure 17:**
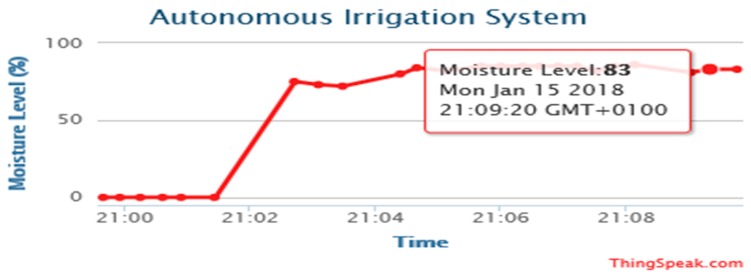
Moisture levels against time obtained from http://www.thingspeak.com (API key = FE4H530DNA2NJQXB).

**Figure 18:**
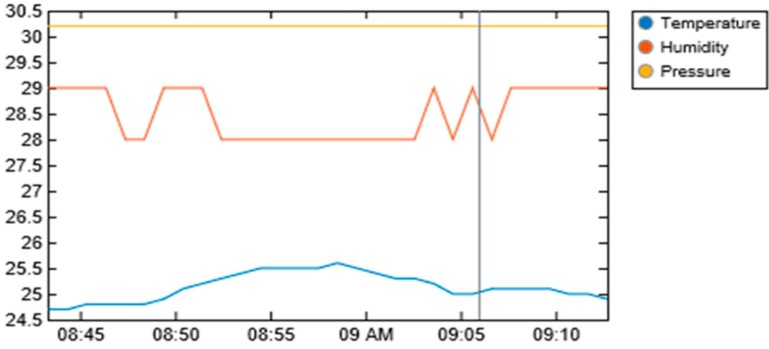
Variation of soil temperature, humidity, and pressure measurements obtained from http://www.thingspeak.com (API key = FE4H530DNA2NJQXB).

**Figure 19:**
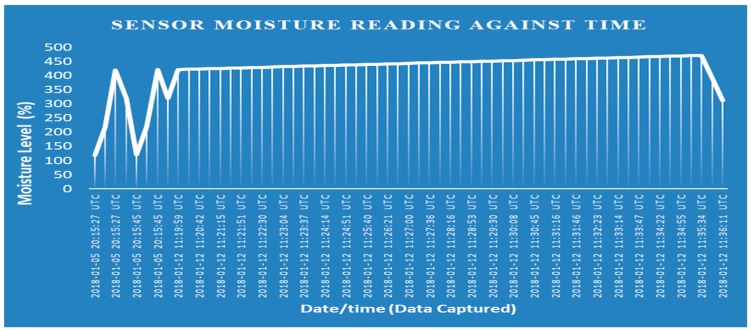
Soil moisture values against time.

Figure 14, Figure 15, Figure 16 and Figure 17 illustrate the calibrated soil moisture values against time. These values are obtained from the sensor reading of the implemented prototype from the website, www.thingspeak.com in real time, using the key value of the application programming interface (API). Figure 18 shows the variation of temperature, humidity, and pressure in the tested soil as reported by the www.thinkspeak.com analytic and visualization website. We can observe that, in Figure 19, the soil status (dry or moist) determines the supply of the water to the plants at different time intervals. As the water content increases, and becomes higher than the threshold values (between 100% and 400% volumetric water content), the supply of water stops, thus preventing the supply of excess water to the plants, which can damage or harm their roots and expose them to the environment. The designed system drastically minimizes water loss and prevents plant damage, which is due to the high-water content of its roots or leaves. Below 100%, the soil moisture is low, and the plant will dry. The water pump is turned ON. Above 400%, the moisture exceeds the threshold limit and the water pump is turned OFF. This shows that the monitoring and control of water content in farmland, plays a vital role in improving the irrigation system of farming and ensuring a significant increase in agricultural productivity, with minimal or no effort from the farmer.

### 4.2. Performance Evaluation on Loamy and Sandy Soils

**Figure 20:**
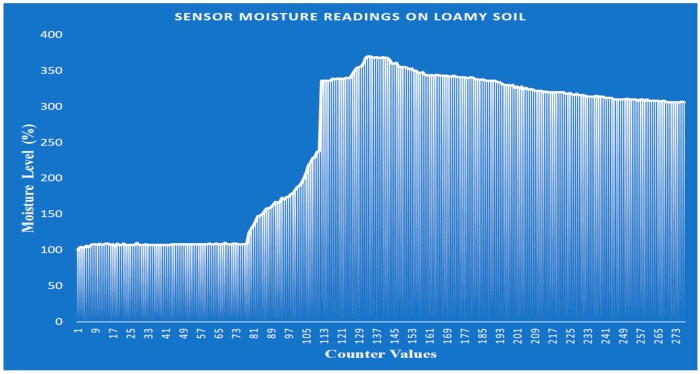
Soil moisture level against counter values on loamy soil.

Loamy and sandy soils are selected for performance evaluation, owing to the fact that they are prominent types of soil that are suitable for autonomous irrigation farming. The test was conducted by setting the system counter at 0 to proceed to 300, and various moisture readings were taken with different counter values, while the device was in operation. Figure 20 illustrates the performance of the loamy soil in terms of moisture content. It can be observed that, as the water is supplied to the soil, the soil tends to absorb the water slowly (i.e., a soil moisture level value of approximately 100%), which is due to the water-retaining capability of loamy soil, and at a particular point (i.e., from the moisture level 300 to 350 percent), it retains water, causing the water pump to steadily switch off to stop the further supply of water. This demonstrates that the implemented prototype can perform satisfactorily when deployed in irrigated farmland enriched with loamy soil. Furthermore, in deploying the system in loamy soil farmland, the cost of electricity consumption is minimized by closing the water pump when the moisture level exceeds 400%. This also control the supply of water to plants and prevent the roots from being exposed to the surrounding environment, resulting in higher crop yield.

**Figure 21:**
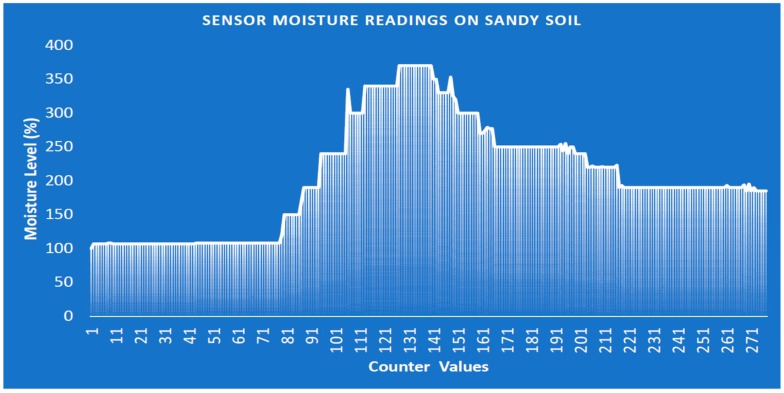
Soil moisture level against counter values on sandy soil.

Figure 21 illustrates the performance of sandy soil in terms of moisture content. As the water is supplied to the dry sandy soil, the water tends to be quickly absorbed by the soil, due to the poor water-retaining capability of the soil. The moisture level is higher at the counter values of 81 to 161. However, the moisture level significantly drops to below 200%, causing the moisture content level to decline significantly with time. The monitoring and control of sandy soil require a greater consumption of electricity to power the water pump, in order to maintain the moisture level status at normal (i.e., between 100% and 400%). This approach is implemented to help inform farmers in real-time that irrigation is required. These findings are of immense assistance to farmers and decision-makers in the proper monitoring and control of agricultural farm products for sustainable development.

### 4.3. Cost Analysis

Table 1 shows the list of items, and their descriptions that are used to design and implement the device prototype. The cost analysis is expressed in U.S. Dollar (USD). These cost patterns are subject to vary from time to time, due to fluctuations in the global market exchange rate [36]. The cost analysis provided excludes the cost of shipping, value added tax, and labor. It can be observed that the total cost of the complete prototype is valued at $79 USD, which indicates a very low-cost device that is affordable and can be utilized by farmers with low income, compared to the approach presented by the authors in [18].

## 5. Conclusions

This article presents a low-cost autonomous sensor interface for the design of an IoT-based smart irrigation monitoring and control system. A real working prototype was designed and implemented. The main objective of this work was to enable farmers to have autonomous monitoring and control of remote farmland, to generate an increase in crop production. This study used a moisture sensor to measure the water content in the soil, a water pump to supply the required amount of water to the plants, and a WiFi module to make the sensed data accessible through the Internet. The web server serves as the main base station for the storage of sensor readings. The data, stored on the web server, were rigorously analyzed. The sensor measurements were transmitted to the Internet in real time. Based on the acquired results, a farmer can precisely monitor the water content of the soil in his/her farmland. The performance evaluation of the system is reliable, in terms of the captured data, speed, accuracy, and integrity of the sensed data that is obtained from the sensors. Based on the descriptive nature of the plotted graphs, a farmer can determine whether a plant is in good condition, in terms of the water content of the soil beneath it. In real time, the system notifies farmers of any inadequate or excess water supply to the farm, as the water pump is switched on or off, depending on the moisture level. The proposed low-cost system can be implemented on a large-scale agricultural farm, to simplify and eliminate the unnecessary stress a farmer normally undergoes in supplying water to his/her farmland. The proposed system, and presented experimental results, will be of immense assistance to farmers and decision-makers in the proper monitoring and control of agricultural farm products.

Future work will consider the use of a field programmable gate array (FPGA) and system-on-chip (SoC)-based architecture, namely, the Xilinx Zynq 7000 FPGA-SoC device using the Python programming language for Zynq devices. This will enhance high performance device monitoring and control when deployed to large agricultural farmland.

## Figures and Tables

**Table 1:** Cost analysis of the implemented device prototype.

S/No.	Item Description	Unit Quantity	Unit Price (U.S. Dollar)	Amount (U.S. Dollar)
1.	Arduino Uno Board	1	12.3445	12.3445
2.	Generic EPS-01 ESP8266 2.4-GHz WiFi Module for Arduino	1	9.25134	9.25134
3.	16 × 2 LCD Blue Screen Microcontroller Development Support for Arduino	1	3.38097	3.38097
4.	LM393 Soil Moisture Sensor	1	2.20438	2.20438
5.	Electrolytic capacitor (20 pcs)	1	5.23540	5.23540
6.	Resistors 1 kΩ (50 pcs)	1	6.88868	6.88868
7.	12-V Relay Module External Trigger Delay Adjustable	1	7.14219	7.14219
8.	Diodes 10 mn RGB LED Module Light Emitting Diode for Arduino	3	2.22918	6.68754
9.	Generic PCs Water Pump High Quality DC 12 V 3.8 m, Magnetic Electrical Centrifugal Hotsel	1	14.8327	14.8327
10.	Breadboard and Jumper Cables.	1	4.13321	4.13321
11.	DHT 11 Digital Temperature, Humidity Sensor Module for Arduino	1	1.29507	1.29507
12.	Generic AC 220 V to 12 V DC step down Power Supply Module for Arduino	1	5.89671	5.89671
Total		14	74.83433	79.29263
